# The Apoptotic Effect of *Trichinella spiralis* Infection Against Experimentally Induced Hepatocellular Carcinoma

**DOI:** 10.31557/APJCP.2021.22.3.935

**Published:** 2021-03

**Authors:** Fawzya A Elhasawy, Dalia S Ashour, Ayman M ElSaka, Howaida I Ismail

**Affiliations:** 1 *Department of Medical Parasitology, Faculty of Medicine, Tanta University, Egypt. *; 2 *Department of Pathology, Faculty of Medicine, Tanta University, Egypt. *

**Keywords:** Trichinella spiralis, hepatocellular carcinoma, apoptosis, Bcl-2

## Abstract

**Background:**

Hepatocellular carcinoma (HCC) is the sixth most common type of cancer. Prognosis of HCC remains unsatisfactory. Therefore, developing new therapeutic modalities is still mandatory. Tumor biotherapy is a novel concept developed as a therapeutic strategy for cancer treatment. There is a similarity between the regulatory mechanism of *Trichinella spiralis* nurse cell formation and tumor cell apoptosis signal regulation.

**Objectives:**

Induction of apoptosis by *T. spiralis* can represent a new strategy for tumor treatment.

**Methods:**

Experimental animals were divided in four groups; negative control (GI), *T. spiralis* infected (GII), induced HCC (GIII) and HCC then infected with* T. spiralis *(GIV). The apoptotic effect of *T. spiralis* infection was assessed by histopathological and immunohistochemical staining of B-cell lymphoma 2 (Bcl-2).

**Results:**

We found higher survival rate of rats and decreased weight of their livers with no nodules in HCC- *T. spiralis* group as compared to HCC group. Improvement of the dysplastic changes and increased apoptotic bodies which was confirmed by decreased expression of Bcl-2 reported in HCC- *T. spiralis* group.

**Conclusion:**

*Trichinella*-induced apoptosis can be a contributing mechanism of the anti-tumor effect of *T. spiralis* infection. Our results showed a certain level of decreased progression of the tumor in HCC-*T. spiralis* group as indicated by increased rate of apoptosis and subsequently had a positive impact on the survival of rats.

## Introduction

Hepatocellular carcinoma (HCC) is the sixth most common type of cancer with about 9.2% of all cancer-related mortalities (Ferlay et al., 2010). HCC is one of the least curable malignancies (Parkin et al., 2005). Despite the presence of multiple treatment options for malignancy including chemotherapy, radiotherapy, and surgery but all these traditional methods have many side effects and only a limited success (Kumari et al., 2018). Moreover, some unresolved issues arise during treatment such as killing of the normal cells, damages of the immune system, drug-toxicity, and drug-resistance (Oldham and Dillman, 2009). HCC prognosis remains unsatisfactory, and new therapeutic modalities are still mandatory. 

Tumor biotherapy is a novel concept developed as a therapeutic strategy for cancer treatment aiming to suppress or eliminate tumors using biological agents. It includes the use of cytokines, monoclonal antibody, growth and differentiation factors, cancer gene therapy, and anti-tumor bioactive materials (Oldham and Dillman, 2009; Torre et al., 2015).

Progressively, experimental studies have demonstrated that parasitic infections can suppress tumors via different mechanisms. For example, *Toxoplasma gondii* lysate antigen and *Toxocara canis* egg antigens were shown to induce a significant increase of anti-angiogenic factors and inhibit tumor growth in experimental models (Darani et al., 2009; Pyo et al., 2010). The anti-neoplastic effects of *Trichinella *have been described in vitro such as in murine ascitic hepatoma cell (H22), hepatoma carcinoma cell (Hepa1-6) and murine forestomach carcinoma cell (MFC) (Zhang et al., 2009). The anti-tumor effect of human hydatid cyst fluid has been documented on the colon carcinoma growth (Berriel et al., 2013). Infection with *Trypanosoma cruzi* decreased the incidence of induced colon cancer via induction of vigorous and long-lasting Th1 immune response (Oliveira et al., 2001; Rankin et al., 2003). 

Despite the different anti-tumor mechanisms that had been described in several parasites, *Trichinella spiralis* is unique in its effect. It produces cytokines that can inhibit tumor growth. Additionally, *T. spiralis* infection causes a variety of changes in the skeletal muscle cells during nurse cell formation including muscle cell differentiation, apoptosis and arrest of the infected cell in the cell cycle at G2/M (Jasmer, 1993). It was reported that crude *T. spiralis *extract arrested the growth of human chronic myeloid leukemia cell line (K562) at G1 phase and hepatoma cell line (H7402) at S phase (Wang et al., 2009). Moreover, the mechanism by which* T. spiralis *and its extract inhibit tumor growth and induce apoptosis of tumor cells was suggested to involve regulation of apoptosis-related genes and triggering a mitochondrial pathway or death receptor pathway (Wang et al., 2009). Infection with *T. spiralis* involves the expression of c-Ski protein (a tumor suppressor protein), in cooperation with signaling pathway genes such as p53 (apoptosis gene expression), SMAD2 and SMAD4 (Boonmars et al., 2005; Wu et al., 2006).

Therapeutic strategies to induce apoptosis or inhibit anti-apoptotic signals may help to reverse, delay, or prevent oncogenesis (Sharaf-el-dein et al., 2011; Vasilev et al. 2015). Therefore, the apoptotic effect of parasites or their secretory products can be employed as a new biological era in cancer treatment. Our study aimed to investigate the apoptotic effect of *T. spiralis* infection as a possible mechanism of its anti-tumor effects in HCC in vivo. 

## Materials and Methods

*Animals and parasite*

This study had been conducted on 95 Swiss albino rats (6-8 weeks old, weighing 85-120 gm at the time of experiment). Animals were housed in appropriate cages and maintained on commercially available standard pellets and water ad libitum, according to the institutional and national guidelines. The strain of *Trichinella* was isolated from infected pork meat obtained from Cairo abattoir, genotyped as* T. spiralis* by the European Union Reference Laboratory for Parasites, Superior Institute of Health, Rome, Italy and maintained in the Laboratory of Medical Parasitology Department, Tanta Faculty of Medicine by consecutive passages through laboratory rats and mice. Infection was done orally with 1,000 *T. spiralis* larvae/ rat according to Dunn and Wright (1985).

*Experimental design*

Rats were divided into 4 groups: group I (15 rats): uninfected untreated rats (control group), group II (20 rats): *T. spiralis*-infected rats (*T. spiralis* group), group III (30 rats): rats with induced hepatocellular carcinoma (HCC group) and group IV (30 rats): rats with HCC then infected with *T. spiralis *(HCC-*T. spiralis* group). All rats were housed at the same time. HCC was induced in groups III and IV. Then, after the onset of tumor formation (three months from the beginning), rats from group IV were infected with *T. spiralis*. At this time, *Trichinella* infection was induced in group II. 

*Induction of HCC*

Chemically induced HCC was performed by intraperitoneal injection of a single dose of diethylnitrosamine (DEN) at a dose of 200 mg/kg body weight followed by subcutaneous injections of carbon tetrachloride (CCL4) at a dose of 3 mL/kg body weight (twice /week) for three months. HCC was confirmed by increased serum alpha fetoprotein level (data not shown) and by histopathological examination of liver of two sacrificed rats. Carbon tetrachloride was continued throughout the experiment to maintain its carcinogenic effect on the rats (Saad El-Din et al., 2018).

The used chemicals were prepared as follows: 

• Diethylnitrosamine 

(CAS 55-18-5, N-nitrosodiethylamine) (Sigma Aldrich, Egypt): one mg DEN dissolved in 15 mL phosphate buffer saline (PBS 0.67%, w/v) on the day of injection. It acts as initiator for HCC in animal models because of its DNA alkylating and genotoxic effects and it produces reactive oxygen species resulting in liver cell injury (Subramanian et al., 2007; Singh et al., 2009).

• Carbon tetrachloride (CAS 56-23-5) (Sigma Aldrich, Egypt): One ml CCL4 dissolved in olive oil to a final volume of 10 ml (0.01%, v/v). This solution was prepared freshly at least once a week and it must be protected from light. It acts as a promotor of HCC as it is metabolized by the liver and produces free radicals which reduce the hepatic antioxidants and damage macromolecules leading to tumor genesis (Weber et al., 2003). 

Close observation of rats was performed twice weekly for signs of morbidity, weight reduction and any external tumor appearance. Survival rate was recorded throughout the study.

*Collection of specimens*

Rats from group II were scarified at 20, 30 and 40 days p.i. (five rats each). Rats from groups III and IV were scarified 20, 30 and 40 days from the onset of tumor formation (five rats each). Rats from the control group I were sacrificed on the corresponding time periods (five rats each time). The liver was excised with sharp dissecting scissors and weighed by electronic microbalance and the wet liver weight was recorded, examined macroscopically for any color changes or presence of areas of hemorrhage and necrosis then placed in 10% neutral buffered formalin for further studies. 

*Histopathological and immunohistochemical analysis*

Liver specimens were processed and subsequently embedded in paraffin wax, sectioned and stained with haematoxylin and eosin (H&E) (Feldman and Wolfe, 2014). Assessment of histopathological changes in the liver tissues regarding hepatocytes arrangement, nucleus size and shape and inflammatory changes was done. 

For immunohistochemical staining of B-cell lymphoma 2 (Bcl-2) expressing cells: paraffin sections of liver specimens were stained using an immunoperoxidase method. Briefly, tissue sections were de-paraffinized, hydrated and treated with 3% H_2_O_2_ to eliminate endogenous peroxidase activity, incubated in citrate buffer (pH 6.0) for antigen unmasking. Then, the sections were incubated with Bcl-2 monoclonal antibody (Dako, Cambridge, UK) in dilution 1:200 then, washed twice with PBS followed by incubation with avidin-biotin complex-conjugated secondary anti-mouse antibody (1:1,000) and streptavidin-conjugated peroxidase. Diaminobenzidine tetrahydrochloride (DAB) was used as a chromogen for 5 min, and haematoxylin was used as a counterstain. Localization of Bcl-2-expressing cells within the nucleus and/or cytoplasm was assessed. A section was categorized either positive or negative in a blinded manner according to the presence or absence of brown staining in cell cytoplasm and/or nucleus (Liang et al., 2019). B cell lymphoma cell line was used as a positive control for Bcl-2. Grading of immunohistochemical staining of Bcl-2-expressing cells was done as follows. Percentage of positive cells (PP %) was scored as 0 for < 5%, 1 for 5-25%, 2 for 25-50%, 3 for 50-75% and 4 for > 75. The staining intensity (SI) was scored as 1 for weak, 2 for moderate and 3 for intense. The immunoreactivity score (IRS) was evaluated by multiplying the percentage of positive cells and the staining intensity. If the IRS was less than 6, it means low expression and if IRS was equal or more than 6, it means high expression (Chan et al., 2007; Hussein et al., 2002).

*Statistical analysis*

Quantitative values of the measured parameters were expressed as mean ± standard deviation (SD). The data were analyzed by one way- ANOVA with tukey post hoc test to determine the significance of differences between groups. The probability of significant differences was determined by chi-square test for the immunohistochemical results. The difference was considered statistically significant when P < 0.05, highly significant when P < 0.01 and not significant when P ˃0.05. Statistical Package for Social Sciences (SPSS) version 14.0 was used.

## Results

*Survival rate and general condition of rats*

The survival rate of negative control rats (group I) and rats infected with *T. spiralis* (group II) was 100%. While that of HCC (group III) was 63%. On the other hand, the survival rate of HCC-*T. spiralis* group (group IV) was 90 %. Only rats of HCC group (group III) and HCC- *T. spiralis *group (group IV) showed ulcers at the site of CCL4 injection. 

*Mean body weight of rats*

The mean body weight of rats of the four groups was increased with time with a highly significant increase in the same group at each duration while rats of HCC group showed reduction of weight in at 40 days from the onset of tumor formation as compared to other groups with statistically insignificant difference (Table 1).

*Gross picture and the mean wet weight of the liver*

**Figure 1:**
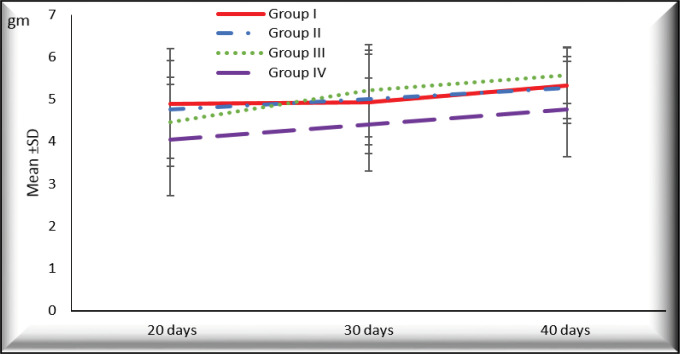
The Mean Values of Liver Weight of Rats of Different Groups at Different Durations

**Figure 2:**
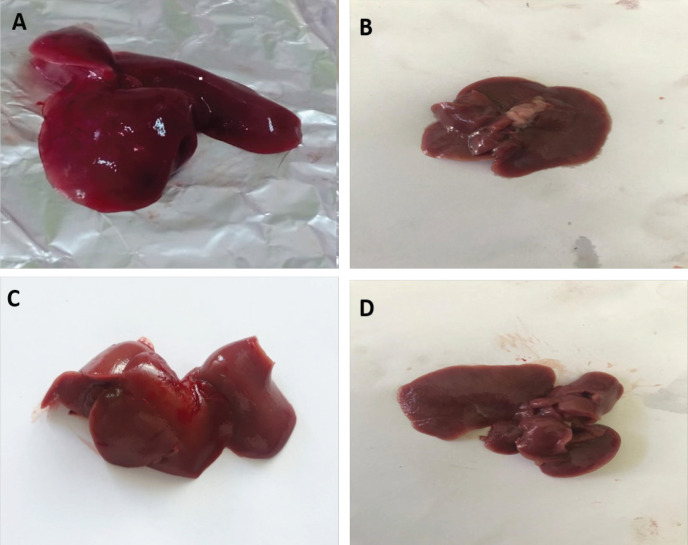
Photomicrograph of the Liver Showing Its Gross Appearance. A, outer surface; B, inner surface of liver from the negative control rats (group I); C, outer surface; D, inner surface of liver from rats infected with *T. spiralis* (group II) showing smooth surface, normal color with no macroscopic changes (no hemorrhage, no necrosis, and no masses).

**Figure 3:**
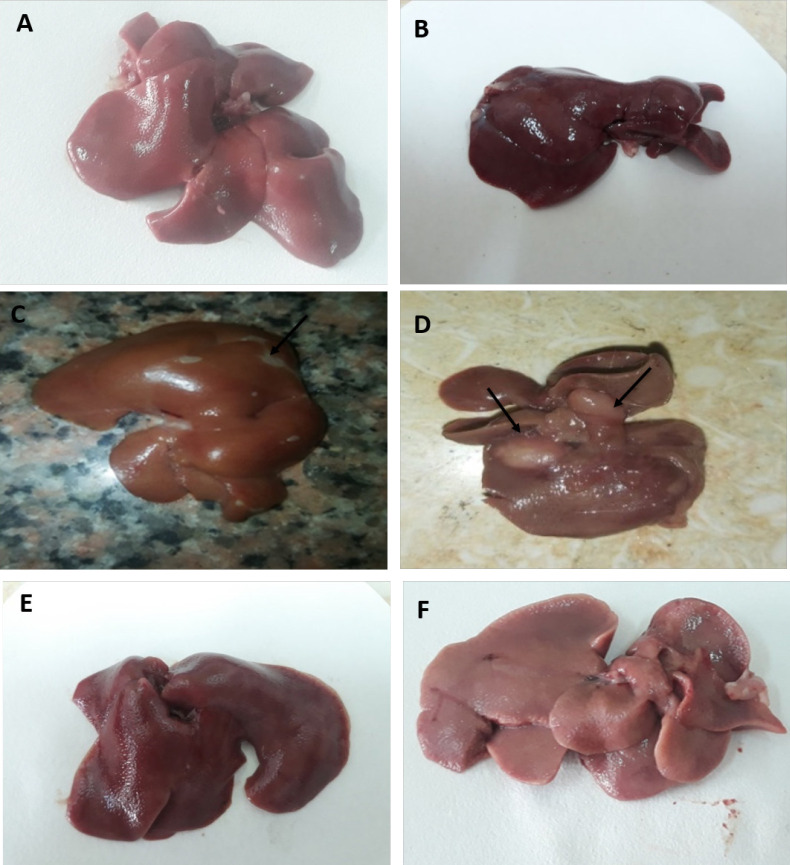
Photomicrograph of the Gross Appearance of Liver of Rats with HCC (group III) (A-D) and HCC- *T. spiralis *group (group IV) (E-F) showing: (A and B) 20 days from the onset of tumor formation showing dark color of liver and the surface was not smooth, (C and D) outer and inner surfaces of liver 40 days from the onset of tumor formation showing dark color of the liver and the surface was not smooth with some nodules (arrows) and (E-F) outer and inner surfaces of liver showing its gross appearance with slightly darker color and the surface was not smooth

There was increased liver weight in different groups with time with the highest values in rats with HCC (group III) at 30 and 40 days from the onset of tumor formation (5.207± 1.090 and 5.567±0.666, respectively) compared to (4.403±1.102 and 4.767±1.124, respectively) in HCC- *T. spiralis* (group IV). There were statistically insignificant differences between the groups at different durations (P > 0.05) (Figure 1). Liver surfaces was smooth and normal color with no macroscopic changes (no hemorrhage, no necrosis, no masses) in negative control rats (group I) and *T. spiralis* group (group II) in different durations (Figure 2; Supplement and supporting data). Meanwhile, rats of HCC group (group III) showed darker liver color and the surface was not smooth and some of them showed nodules that appeared 40 days from the onset of tumor (Figure 3A-D; Supplement and supporting data). Liver from rats with HCC- *T. spiralis *(group IV) was slightly darker than the normal liver and the surface was not smooth with no nodules (Figure 3E-F; Supplement and supporting data). 

*Histopathological changes of the liver*

**Figure 4:**
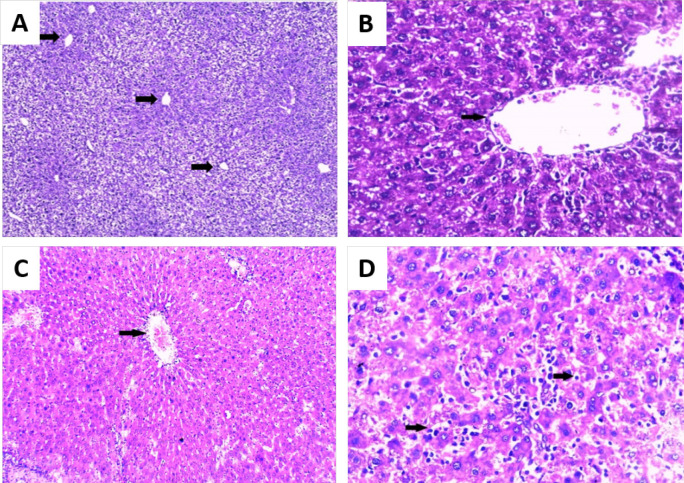
Photomicrograph of Liver Sections (H&E): A- B: negative control rat (group I) showing normal hepatic architecture (arrows showing central veins); A: (×100) and B: ×200) and C-D: *T. spiralis* infected group (group II); C: showing normal hepatic architecture (arrow showing central veins) (×100) and D: showing apoptotic bodies (arrows) (H&E ×250).

The liver tissues of negative control (group I) and rats infected with *T. spiralis *(group II) displayed a well-defined normal hepatic architecture where polygonal hepatocytes showed normal shapes of nuclei and cytoplasmic walls throughout the experiment. They were arranged in plates that radiate from central vein towards portal tracts. Hepatic plates are separated by thin sinusoids with normal sinusoidal size (Figure 4A-C). In addition, some apoptotic bodies appeared in the liver sections of rats infected with *T. spiralis* (group II) on 30 and 40 days p.i. (Fig. 4D). 

**Figure 5:**
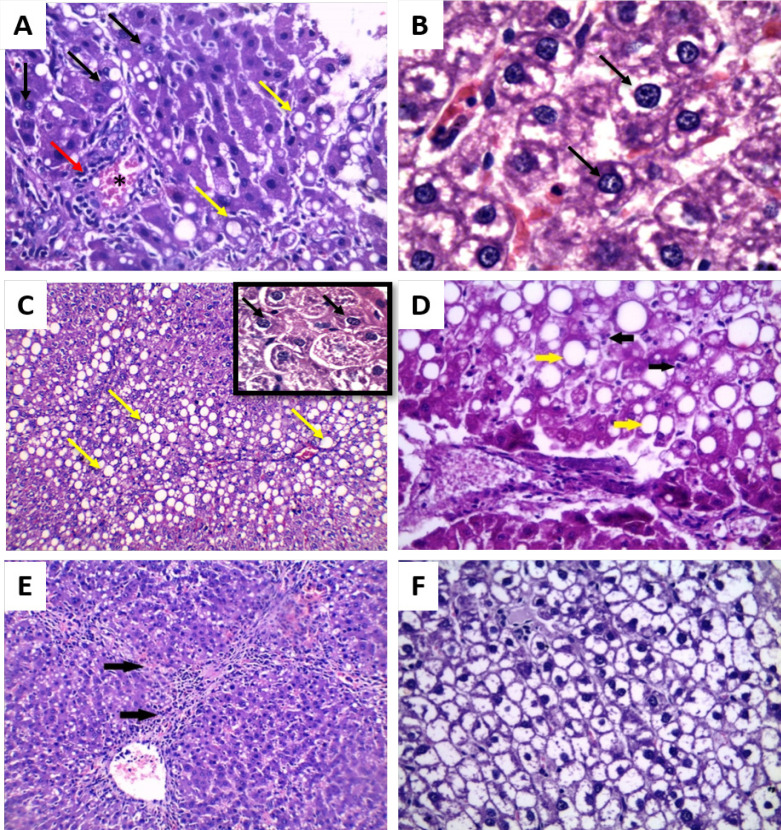
Photomicrograph of Liver Sections of Rats with HCC (group III) (H&E): A: 20 days from the onset of tumor formation showing dysplasia such as increase nucleus size, nucleolus start to appear (black arrow), congestion (asterisk), fatty degeneration (yellow arrow) and infiltration with inflammatory cells mainly lymphocytes (red arrow) (×250), B: 20 days from the onset of tumor formation showing prominent nucleolus, double nuclei (black arrow), clumped chromatin and increased nuclear cytoplasmic ratio (H&E ×400), C: 30 days from the onset of tumor formation showing pleomorphism (multiple prominent eosinophilic nuclei, irregular nuclear membrane (black arrows) and steatosis (yellow arrows) (×250) with insit (×400), D: 30 days from the onset of tumor formation showing few apoptotic bodies (black arrows), steatosis (yellow arrows) (×400), E: 40 days from the onset of tumor formation showing bridging fibrosis (black arrows) and nodules of malignant liver cells (× 250) and F: 40 days from the onset of tumor formation showing clear cell HCC (× 400)

On the other hand, rats with HCC (group III) showed dysplastic changes in the hepatocytes such as increased nuclear size and the nucleolus started to appear 20 days from the onset of tumor formation. Distorted liver pattern and thickened trabeculae were observed. In addition, there were congestion, mild fatty degeneration and infiltration with inflammatory cells mainly lymphocytes (Figures 5A and B). After 30 days from the onset of tumor formation, they revealed an increase of dysplastic changes and increase of nucleus size and prominent nucleolus (i.e. pleomorphism). Steatosis and fatty degeneration presented as widely distributed lipid droplets in the liver parenchyma were observed. Few apoptotic cells were detected (Figures 5C and D). Irregular arrangement and distorted pattern of the liver cells were detected 40 days from the onset of tumor formation. More increase of dysplastic cells such as mitotic figures and more increase in the nuclear/ cytoplasmic ratio were observed. HCC was presented in some liver sections as nodules of malignant cells separated by bridging fibrosis consisting of collagen fibers with a characteristic lamellar pattern (Figure 5E). Clear cell variant of HCC was also detected. The cells showed clear cytoplasm that contains glycogen and fat vesicles arranged in a trabecular pattern (Figure 5F).

**Figure 6:**
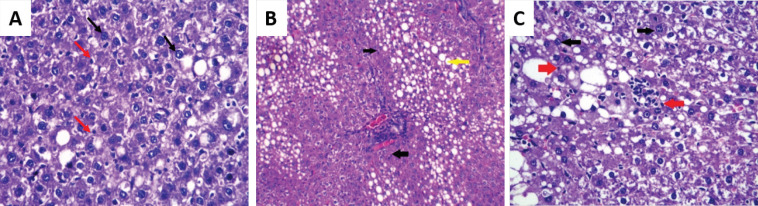
Photomicrograph of Liver Section of HCC- *T. spiralis* group (group IV) (H&E): A: 20 days from the onset of tumor formation showing apoptotic bodies (red arrows) and dysplastic nuclear changes (black arrows) (× 250), B: 30 days from the onset of tumor formation showing irregular arrangement of hepatocytes, fatty degeneration (yellow arrow) and apoptotic bodies (black arrows) (×100) and C: 40 days from the onset of tumor formation showing apoptosis (red arrows), nuclear dysplasia and pleomorphism (black arrows) (× 400)

While liver sections from rats of HCC-*T. spiralis *group (group IV) revealed the appearance of some apoptotic bodies 20 days from the onset of tumor formation in addition to the presence of some dysplastic changes as described before (in group III) (Figure 6A). They showed a certain level of improvement when compared to the HCC rats in the form of increased number of apoptotic bodies among the dysplastic cells with time (at 30 and 40 days from the onset of tumor formation) in addition to the irregular arrangement of hepatocytes and fatty degeneration (Figures 6B and C). 

*Immunohistochemical staining of Bcl-2-expressing cells*

**Figure 7:**
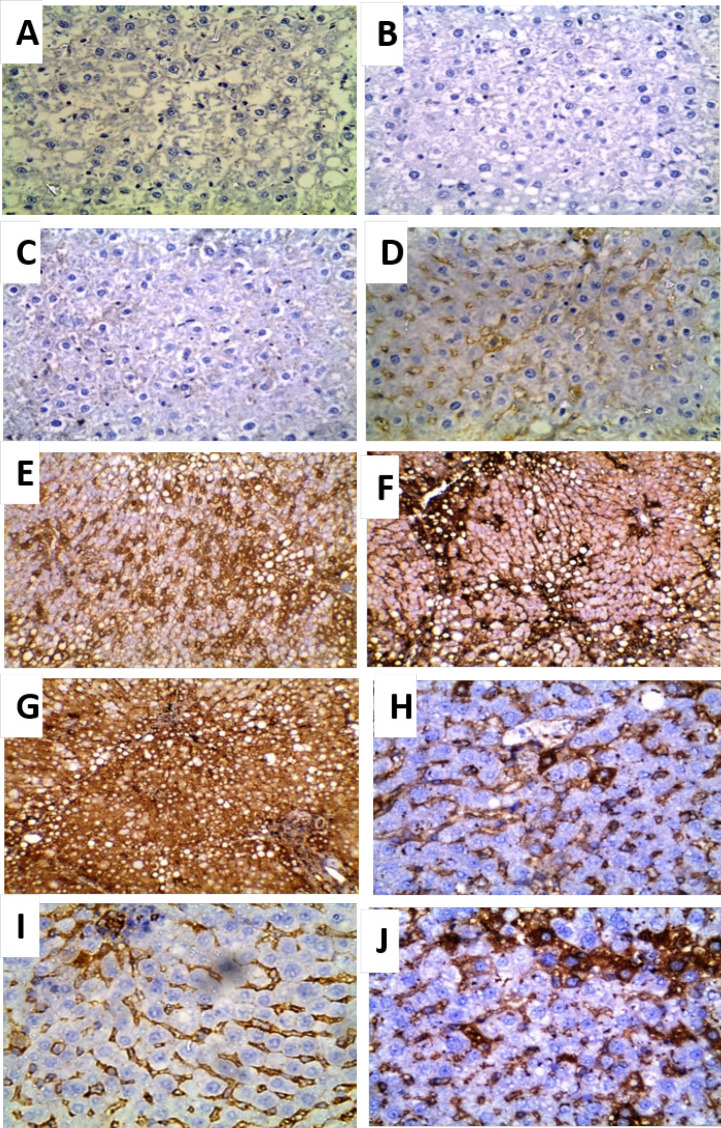
Photomicrographs of Immunohistochemical Localization of Bcl-2 in Liver Sections (×400): A: negative control rats (group I) showing negative expression of Bcl-2, B-D: *T. spiralis* infected rats (group II); B: 20 days p.i. showing negative expression of Bcl-2 (IRS=zero), C: 30 days p.i. showing weak expression of Bcl-2 (IRS=1), D: 40 days p.i. showing moderate expression of Bcl-2 (IRS=2), E- G: HCC group (group III) showing high expression Bcl-2; E: 20 days from the onset of tumor formation (IRS=4), F: 30 days from the onset of tumor (IRS=6) and G: 40 days from the onset of tumor formation (IRS=12) and H-J: HCC- *T. spiralis* group (group IV) showing weak expression Bcl-2; H: 20 days from the onset of tumor formation (IRS=3 ), I: 30 days from the onset of tumor formation (IRS=2) and J: 40 days from the onset of tumor formation (IRS=1).

A semiquantitative analysis of Bcl-2-expressing cells of liver sections from negative control rats (group I) showed negative expression of Bcl-2 (Figure 7A) while rats of *T. spiralis* group (group II) showed negative, weak and moderate expression of Bcl-2 on 20, 30 and 40 days p.i., respectively (Figure 7B- D) with a statistically insignificant increase.

In liver sections from rats with HCC (group III), high expression of Bcl-2 was observed in different durations with a highly significant increase only on 40 days as compared to that of 20 days from the onset of tumor formation (P< 0.01) (Figure 7E- G). In contrast, *Bcl-2*-expressing cells in HCC-*T. spiralis* rats (group IV) showed weak expression of *Bcl-2* (Figure 7H- J). There was a highly significant reduction in Bcl-2-expressing cells on 40 days as compared to 20 days (P< 0.01).

**Figure 8:**
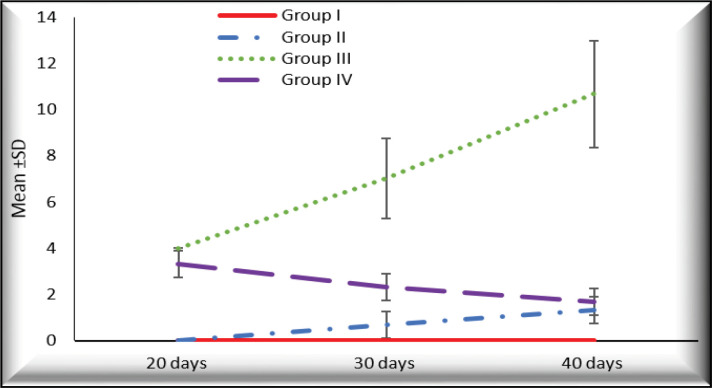
The Mean Values of Immunoreactivity Score (IRS) at Different Durations in Different Groups

There was a highly significant increased expression of *Bcl-2* in group III as compared to both groups I and II at different durations and on 30 and 40 days from the onset of tumor formation as compared to group IV (P< 0.001). While group IV showed a highly significant increased expression of *Bcl-2* only on 20 days from the onset of tumor formation as compared to both groups I and II (P<0.001) (Figure 8).

## Discussion

HCC is a well-known strong tumor with a 5-year survival time less than 5% (Forner et al., 2012). The currently available methods are effective only in some patients and associated with postoperative mortality and high risk of recurrence (Portolani et al., 2006; Jo et al., 2016; Kumari et al., 2018). Therefore, new treatment options are urgently needed.

*Trichinella spiralis* nurse cell formation is a complex process and involves de-differentiation and cell cycle arrest of infected muscle cells (Wu et al., 2008). The infected muscle cells show morphological features of apoptosis such as nuclear fragmentation, mitochondrial swelling and degradation (Dabrowska et al., 2008). These changes are associated with up-regulation of apoptosis factors that mediated a mitochondrial pathway such as Bcl-2 associated protein X (BAX) and caspase 9 and a death receptor pathway such as tumor necrosis factor-alpha (TNF-α), caspase 8 and caspase 3 (Boonmars et al., 2005). 

In addition to the cell cycle arrest of the infected muscle cells, *T. spiralis* infection up-regulates the expression of some apoptosis-related genes such as SMAD2 and SMAD3 that act as tumor suppressor genes (Wu et al., 2008; Samanta and Datta, 2012). In other words, the nurse cell formation apoptotic pathways may be activated by anti-tumor genes that can suppress cell proliferation or induce apoptosis of the tumor cells (Wang et al., 2009). 

Because of the similarity between nurse cell formation regulatory mechanism and tumor cell apoptosis signal regulation, infection with *T. spiralis* can induce apoptosis of HCC and represents a new strategy for its treatment (Wu et al., 2008; Babal et al., 2011; Marquardt and Edlich, 2019). Therefore, we aimed to investigate the apoptotic effect of *T. spiralis* infection as a potent inhibitor for tumor growth in HCC model. 

In the present study, only rats with HCC either infected with *T. spiralis* or not (group IV and group III), respectively were suffered from skin ulcers at the site of drug injection. It is suggested that the ulcers might be due to subcutenous injection of CCL4. Carbon tetrachloride is toxic on contact with the skin and it causes some degenerative changes in the epidermis, slight karyolysis, junctional separation and cellular infiltration in the dermis (Kronevi et al., 1979; del Río et al., 2014).

The mean body weight of HCC group (group III) was less than the other three groups with no stastistically significant reduction that might be related to the toxic effect of CCL4 and DEN and the decrease of the appetite. Similar studies related to HCC reported body weight reduction and tissue destruction (Singh et al., 2018; Badr El-Din et al., 2020) as compared to the control group. 

Regarding the survival rate, rats with induced HCC (group III) reported the least survival rate (63%). The deaths of rats in group III were mostly due to the general toxic effects of DEN and CCL4 (JO et al., 2016). While that in rats with HCC- *T. spiralis* (group IV) was 90 % that reflects the protective role of *T. spiralis* infection. In the same context, Molinari and Ebersole (1977) demonstrated that mice with chronic *T. spiralis* infection survived for a significantly longer time and had smaller tumors after application of B16 melanoma cells, compared to animals that were not infected. 

The mean wet liver weight of rats with HCC (group III) and HCC- *T. spiralis* (group IV) was increased with the time of the experiment with higher increase in group III on the 40^th^ day from the onset of tumor formation. This increase might be explained by the effect of DEN on the liver as mentioned by JO et al., (2016) and Singh et al., (2018) who stated that treatment with DEN to induce HCC caused increasing in the liver weight. Meanwhile, the reduced liver weight in group IV as compared to group III suggested that the enlargement of the liver caused by DEN might be diminished by effect of* T. spiralis *infection.

Regarding the gross picture of the liver, HCC (group II) showed darker color and some surface nodules. Trevisani et al., (1993) mentioned that HCC frequently presented with a nodular appearance showing fibrous capsules and septa. However, the gross appearance of HCC is affected by various factors such as tumor size, tumor thrombus in the portal and hepatic veins, intrahepatic metastasis, necrosis and hemorrhage (Kai et al., 2012). Liver of rats with HCC- *T. spiralis* (group IV) showed no surface nodules which indicates the protective effect of *T. spiralis* infection on HCC.

Regarding the histopathological findings, some apoptotic bodies appeared in the liver sections of rats infected with *T. spiralis* (group II) on 30 and 40 days p.i.. Boonmars et al., (2004) found that the expression of mitochondrial apoptosis related genes was elevated in *T. spiralis* infected muscle during encapsulation. The presence of similar apoptotic effects in the liver tissues in our study can be explained by the finding reported by Vasilev et al., (2015) who mentioned that the excretory/secretory products of *T. spiralis* muscle larvae influence the host at a systemic level and for long period. 

In contrast, HCC group (group III) showed histopathological changes indicative for progression of tumor involving more dysplastic cells, distorted liver pattern and no apoptotic bodies were observed. These results coincide with many other studies that investigated the histopathological changes in DEN-induced HCC. Tolba et al., (2015) and Singh et al., (2018) demonstrated that the DEN-induced liver cancer group revealed an irregular cellular structure with divided nuclei, which had lost their spherical shapes. Cui et al., (2018) established a steatohepatitis-HCC mouse model using DEN. They described the appearance of lipid drops in hepatic parenchyma, inflammatory infiltration, hepatocyte ballooning, and destruction of normal hepatic architecture, plate-like growth and abnormal cytological structure of hepatocytes. Dysplastic cells are discriminated from the surrounding normal liver tissue by their morphology, cytoplasmatic staining, nuclear size with an increased nuclear/cytoplasmatic ratio and cellular atypia (Schlageter et al., 2014). Dysplastic foci of HCC are characterized by low rate of apoptosis (Le Bail et al., 1997).

It is well known that resistance to apoptosis is the hallmark of cancer (Wang et al., 2013a). Therefore, the increased rate of apoptosis induced by *T. spiralis* infection resulted in decreased tumor cell survival.

In the present study, we found that the HCC- *T. spiralis *group displayed histopathological changes indicative of apoptosis reaching its highest level 40 days from the onset of tumor formation. Although no previous studies were conducted to investigate the effect of *T. spiralis* infection on HCC *in vivo*, some trials were performed to explain its anti-tumor activity in vitro. Wang et al., (2009) have found that *T. spiralis* adult crude extract had an in vitro anti-proliferative effect on the hepatoma cell line H7402. Moreover, they stated that *T. spiralis* ES products exerted similar anti-tumor activity to live *T. spiralis* infection. Similar results were reported by Wang et al., (2013b). They showed that increased expression of A200711 gene produced an anti-tumor protein by *T. spiralis* that had an apoptotic effect on the human hepatoma cell line (H7402).

The apoptotic effect of *T. spiralis* reported in our histopathological results was confirmed with immunohistochemical staining of *Bcl-2*-expressing cells. Bcl-2 family has emerged as a dominant regulator of apoptosis in cancer cells. The Bcl-2 protein exerts its anti-apoptotic functions by modulating the mitochondrial release of cytochrome c and the interaction of apoptosis activating factors (Apaf-1) with caspase 9 and Bax (Bcl-2 associated X protein) (Hussein et al., 2003).

In the current study, the negative control rats (group I), showed negative expression of *Bcl-2*. Although Bcl-2 family members have an essential role in liver homeostasis, it is not generally expressed in normal liver tissue (Charlotte et al., 1994). While *T. spiralis* group (group II) showed increased expression of *Bcl-2* to show moderate expression 40 days p.i. The same trend of *Bcl-2* expression was reported by Babal et al., (2011). They evaluated the role of *T. spiralis*-induced apoptosis and apoptosis-related factors including Bcl-2 in the process of striated muscle cell transformation. They showed that weak in *Bcl-2* expression at the stage of invasion and moderate expression 45 days p.i.

The cell cycle arrest that occurred during chronic *T. spiralis* infection with subsequent apoptosis in the arrested cells was suggested as a possible mechanism for the anti-tumor activity of *T. spiralis* (Hahm et al., 2007; Moore et al., 2010). Mitochondrial and death receptor signaling pathways are up-regulated in the cytoplasm, with increased expression of mitochondrial apoptosis-related genes and death receptor pathway-mediated apoptosis factors in the basophilic cytoplasm of the infected muscle cells (Boonmars et al., 2005; Wu et al., 2008). 

In contrast, HCC group showed an increase in *Bcl-2*-expressing liver cells reaching its peak 40 days from the onset of tumor formation. These results agree with Lacronique et al., (1996) and Guo et al., (2002) who stated that the development of HCC can be correlated with the increase in *Bcl-2* expression. Moreover, they suggested that Bcl-2 is playing a role in tumor genesis by inhibiting apoptotic death rather than promoting cell proliferation. Many studies reported that a high dramatic increase was detected in the expression of *Bcl-2* in HCC (Fiorentino et al., 1999; Pizem et al., 2001; Hussein, 2004).

The induction of tumor cell apoptosis has been suggested as a new strategy for modern tumor therapy (Kurose et al., 2012). In HCC-*T. spiralis *group, Bcl-2 expression 20 days from the onset of tumor formation was higher than that of 30 and 40 days which indicates that *T. spiralis *apoptotic effect on HCC had increased with time. Liao et al., (2018) mentioned that the extent of the decrease in the tumor growth was different according to the inoculation time after infection with *T. spiralis*. Infection with *T. spiralis *achieved a statistically significant increase in the survival time of mice inoculated with sarcoma 180 tumor cells on the 28 days p.i. and no detectable effect 56 days p.i (Lubiniecki and Cypess, 1975). 

Similarly, Luo et al., (2017) suggested that *T. spiralis *E/S proteins can induce apoptosis in small-cell lung cancer cells. They detected the highest value of the expression of anti-apoptosis genes *Bcl-2 *and livin in cancer cells compared with those co-cultured with *T. spiralis* E/S proteins and increased expression of pro-apoptosis genes *Cyt-C, Apaf-1, caspase-9* and *caspase-3* in the treated cancer cells. 

In the same context, the aim of the conventional therapy of HCC is to induce apoptosis and inhibit tumor proliferation via chemotherapeutic drugs. Compatible with our findings, Deng et al., (2018) proved the apoptotic effect of Corilagin drug that induced the mitochondrial apoptotic and death receptor pathways and resulted in inhibition of HCC cellular proliferation. Moreover, Wang et al., (2018) proved the apoptotic effect of combination therapy sorafenib and bufalin that resulted in inhibition of cell proliferation and regression of HCC when compared with the untreated control group. However, the drug therapy showed poor prognosis of HCC afterward due to the development of drug resistance (Liu et al., 2016).

The apoptotic effect of *T. spiralis* infection against other types of tumors has been reported. Gong et al., (2011) proved the anti-tumor effect of *T. spiralis* crude antigens, ES L1 antigens and tropomyosin in inhibition of myeloma growth. Vasilev et al., (2015) reported that chronic *T. spiralis* infection revealed significantly higher numbers of apoptotic cells in melanoma cells *in vivo* as compared to the control non-infected group. These results agree with the apoptotic effect observed in our study where the number of apoptotic cells in HCC-*T. spiralis* group was higher than that of HCC group.

The anti-tumor effect of some other parasite had been reported. Yang et al., (2013) confirmed the inhibitory effect of *Schistosoma japonicum* recombined protein (rSj16) on murine myeloid leukemia cells and accompanied by decreased expression of *Bcl-2* in rSj16 treated group. Chen et al., (2011) demonstrated the anti-tumor effect of Plasmodium yoelii on Lewis lung cancer that inhibited tumor growth and metastasis and prolonged the survival of mice. 

In contrary, Eissa et al., (2019) observed that mice-treated with autoclaved *T. spiralis* antigen (ATSA) provided a non-significant difference in cancer colon incidence and multiplicity as compared to cancer controls. They suggested that the coincidence of ATSA’s initial immune-stimulant response with the carcinogen-induced inflammatory effect could be responsible for the observed promotion of cancer colon growth. Therefore, it is important to mention that each parasite and/or its products show a unique tumor specificity and the anti-tumor effect can be different according to the tumor cell type. 

The anti-neoplastic impact of *T. spiralis* infection and its derived antigens has been shown by a limited number of studies that focused mostly on the role of chemokines expressed during infection (Kang et al., 2013). Therefore, the exact mechanism of how *T. spiralis* inhibits tumor growth has remained unclear (Lioa et al., 2018). Our current study highlighted the role of apoptosis as an anti-tumor mechanism of *T. spiralis* infection. It is not surprising that the apoptotic pathway may be activated by anti-tumor genes expressed during the cyst formation of *T. spiralis* (Wu et al., 2006; 2008). It was reported that the over-expression of *SMAD3* reduced the level of *Bcl-2 *sensitized hepatocytes (Yang et al., 2006; Alenzi et al., 2010). However, further studies are needed to elucidate the impact of gene therapy as anti-tumor strategy.

In spite that some authors reported that infection with *T. spiralis *rarely leads to significant morbidity (Kociecka, 2000), the infection by *T. spiralis* itself is not an accepted idea to treat diseases for fear of its risk. However, based on the results of our present study, *T. spiralis* should be considered as a potential source of anti-tumor proteins that could be effective and safer than the infection.

In conclusion, we proposed that apoptosis can be a contributing mechanism of the anti- tumor effect of* T. spiralis* infection. Our results showed a certain level of decreased progression of the tumor in HCC-*T. spiralis* group as indicated by increased rate of apoptosis and subsequently had a positive impact on the survival of rats when compared to the HCC group. Further studies are needed with longer periods of follow-up to detect if there is recurrence of HCC or metastasis and to elucidate the responsible protein and gene involved for safe and effective modality of cancer therapy and to decrease resistance to the known chemotherapy.

**Table 1:** Mean Body Weight of Rats in Different Groups at Different Durations

Weight of rats (gm)		Groups												ANOVA	
		Group I			Group II			Group III			Group IV			F	P-value
	Range	95	-	124	76	-	127	90	-	113	80	-	111	1.858	0.155 ^ns^
20 days	Mean ±SD	111	±	9.262	107.9	±	14.456	103.8	±	7.772	100.333	±	9.46		
	Range	111	-	140	92	-	141	100	-	129	92	-	130	2.445	0.080 ^ns^
30 days	Mean ±SD	127.9	±	8.425	127	±	13.84	118.4	±	11.316	116.889	±	11.118		
	Range	127	-	167	116	-	167	116	-	152	118	-	157	1.378	0.267 ^ns^
40 days	Mean ±SD	144.5	±	12.159	149.222	±	16.192	135.889	±	13.393	141	±	15.78		
20-30 days	Differences	16.9	±	4.483	19.1	±	6.887	14.6	±	9.009	16.556	±	8.126		
	Paired Test	<0.001*			<0.001*			0.001*			<0.001*				
20-40 days	Differences	33.5	±	9.022	42.111	±	15.941	33.111	±	18.678	40.667	±	15.158		
	Paired Test	<0.001*			<0.001*			0.001*			<0.001*				
30-40 days	Differences	16.6	±	8.003	23	±	16.47	18.556	±	15.208	24.111	±	9.968		
	Paired Test	<0.001*			0.003*			0.006*			<0.001*				
Post hock test	GI&GII			GI&GIII		GI&GIV		GII&GIII			GII&GIV		GIII&GIV		
P value 20 days	0.913^ns^			0.435 ^ns^		0.144 ^ns^		0.822 ^ns^			0.415 ^ns^		0.891 ^ns^		
P value 30 days	0.998 ^ns^			0.258 ^ns^		0.169 ^ns^		0.341 ^ns^			0.230 ^ns^		0.991 ^ns^		
P value 40 days	0.891 ^ns^			0.569 ^ns^		0.952 ^ns^		0.223 ^ns^			0.625 ^ns^		0.875 ^ns^		

*, Highly significant P < 0.01; ^ns^, non- significant; GI, negative control.; GII,* T. spiralis* group; GIII, HCC group; GIV, HCC- *T. spiralis* group.

## Author Contribution Statement

Study design: DA, the experimental work and collection of data: FE, analysis and interpretation of the results: FE, DA, AE, HI, manuscript preparation: FE, DA, HI. All authors reviewed the results and approved the final manuscript.
